# Natural Inhibitors of Mammalian α-Amylases as Promising Drugs for the Treatment of Metabolic Diseases

**DOI:** 10.3390/ijms242216514

**Published:** 2023-11-20

**Authors:** Aleksandr P. Kalinovskii, Oksana V. Sintsova, Irina N. Gladkikh, Elena V. Leychenko

**Affiliations:** 1 Shemyakin-Ovchinnikov Institute of Bioorganic Chemistry, Russian Academy of Sciences, Moscow 117997, Russia; 2 G.B. Elyakov Pacific Institute of Bioorganic Chemistry, Far Eastern Branch, Russian Academy of Sciences, Vladivostok 690022, Russia; sintsova0@gmail.com (O.V.S.); irinagladkikh@gmail.com (I.N.G.)

**Keywords:** mammalian α-amylases, type 2 diabetes mellitus, obesity, metabolic disorders, α-amylase inhibitors, structural diversity, therapeutic applications

## Abstract

α-Amylase is a generally acknowledged molecular target of a distinct class of antidiabetic drugs named α-glucosidase inhibitors. This class of medications is scarce and rather underutilized, and treatment with current commercial drugs is accompanied by unpleasant adverse effects. However, mammalian α-amylase inhibitors are abundant in nature and form an extensive pool of high-affinity ligands that are available for drug discovery. Individual compounds and natural extracts and preparations are promising therapeutic agents for conditions associated with impaired starch metabolism, e.g., diabetes mellitus, obesity, and other metabolic disorders. This review focuses on the structural diversity and action mechanisms of active natural products with inhibitory activity toward mammalian α-amylases, and emphasizes proteinaceous inhibitors as more effective compounds with significant potential for clinical use.

## 1. Introduction

Diabetes mellitus is a morbid condition that is very common worldwide. Over 90% of cases are type 2 diabetes mellitus (T2D), a disorder marked by defective insulin secretion by pancreatic β-cells and the inability of insulin-sensitive tissues to respond to insulin. According to the International Diabetes Federation, the global diabetes prevalence in 20–79-year-olds in 2021 was estimated to be 10.5% (536.6 million people) and is expected to rise to 12.2% (783.2 million) in 2045 [1]. The number of diabetes cases is growing at an increasing speed. For instance, global diabetes prevalence in 2019 was estimated to be 9.3% (463 million people) and is expected to rise to 10.9% (700 million) by 2045 [2]. Epidemiological studies suggest that many cases of T2D are mainly caused by manageable risk factors, i.e., obesity, low physical activity, and an unhealthy diet; however, genetic predisposition and environmental factors also contribute to the prevalence of diabetes [3]. T2D is seen in all population groups and, lately, is occurring more frequently in children and adolescents [4]. The blood glucose level in patients suffering from diabetes reaches abnormally high values that are toxic to the body. The glucosotoxic effect results in damage to the nerves, vasculature, eyes, kidneys, etc. [5,6].

In humans, four enzymes are responsible for the complete digestion of starch into glucose. Salivary and pancreatic α-amylases (HSA and HPA, respectively, E.C. 3.2.1.1), by virtue of being endohydrolases, cleave intramolecular α-(1→4)-glycoside bonds, producing short linear and branched dextrin. The resulting mixture then undergoes hydrolysis at the non-reducing ends through the action of two small intestine villi exohydrolases: maltase–glucoamylase (MGAM, EC 3.2.1.20 and 3.2.1.3) and sucrose–isomaltase (SI, EC 3.2.1.48). MGAM is a membrane-bound α-glucosidase with two independent N- and C-terminal catalytic domains. They are 40% identical in amino acid sequences and belong to glycoside hydrolase family 31. When consuming starch-rich products, subjects with diabetes and metabolic disorders experience postprandial hyperglycemia. Multiple studies have shown that the inhibition of MGAM and HPA exerts a pronounced therapeutic effect on glycemic control by delaying starch digestion, which enables us to consider them as targets for the treatment of diabetes mellitus, obesity, and other metabolic disorders [7,8,9].

α-Glucosidase inhibitors (AGI), specifically α-amylase inhibitors, are a distinct class of antidiabetic drugs. Natural compounds showing α-glucosidase-inhibitory activity are diverse and numerous; however, this class of drugs is underutilized. The review attempts to summarize the data on the sources, structural diversity, and activity of mammalian α-amylase inhibitors and the practice and prospects for their application in medicine.

## 2. General Aspects of Mammalian α-Amylases

α-Amylases (α-(1→4)-glucan-4-glucanohydrolases, EC 3.2.1.1) are endoglycosidases that catalyze the hydrolysis of α-(1→4)-D-glucosidic linkages in starch, glycogen, and other related polysaccharides. They are ubiquitous in all kingdoms and are among the principal enzymes of carbohydrate metabolism. α-Amylases split starch into maltose, maltotriose, and dextrin. In vertebrates, these products are further hydrolyzed to monosaccharides by intestinal villi enzymes [9].

According to the CAZy database, α-amylases belong to glycoside hydrolase family 13 (GH13) [10]. GH13 proteins are extremely diverse, with only four amino acids conserved throughout the family (a catalytic triad and an additional arginine), but with a similar three-domain tertiary structure. Three distinct evolutionarily related groups among α-amylases have been inferred from amino acid sequences: (i) fungi and yeasts; (ii) plants; (iii) streptomycetes, insects, and mammals [11,12,13]. Animal α-amylases have a high degree of similarity (over 40%) [14], which is greater for mammals (over 84%) (Table 1).

**Figure 1:**
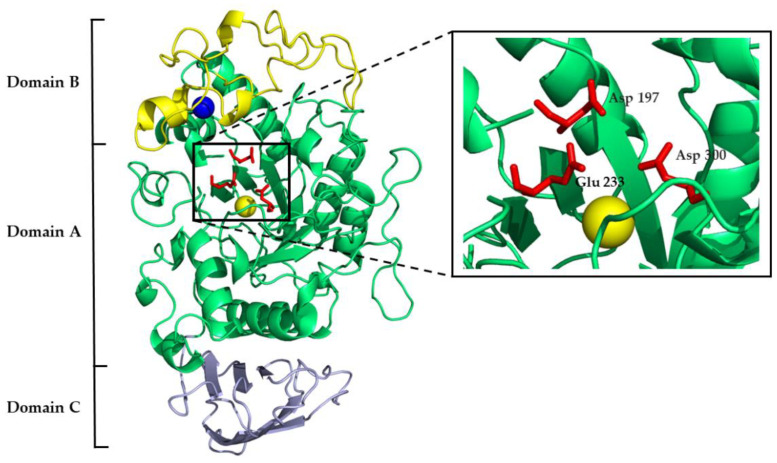
Three-dimensional structure of human pancreatic α-amylase with marked domain architecture. The calcium ion is shown as a blue sphere; the chloride ion is shown as a yellow sphere. The catalytic site residues are shown as red sticks and are magnified in the black box (PDB 1HNY).

Mammalian α-amylases are three-domain proteins stabilized by five disulfide bonds, and they carry three sites that are vital for their functioning: catalytic, calcium-binding, and chloride-binding. The conserved catalytic triad of aspartate, glutamate, and aspartate is contained within domain A (Figure 1), which is a (β/α)8-barrel (i.e., TIM-barrel). Along with other GH13 enzymes, α-amylase catalyzes starch hydrolysis through the classical Koshland double-displacement mechanism where the aspartate acts as a catalytic nucleophile, the glutamate as a general acid/base, and another aspartate stabilizes the transition state. The V-shaped active site cleft between domain A and domain B harbors α-amylase substrates. The chloride-binding site lies in proximity to the active site cleft on domain A and is formed of positively charged arginine and lysine residues. Domain B is built of two antiparallel β-sheets and a long loop with an irregular structure. The calcium-binding site is located between the domain B β3 strand and the α3 helix of domain A, where one calcium ion is coordinated by the ligands of both domains. Domain C is an eight-stranded antiparallel β-barrel of the Greek key motif that is linked to domain A by a flexible polypeptide chain (Figure 1) [15,16,17,18,19].

In animals, α-amylases are found mainly in the pancreas and salivary glands and, to a lesser extent, in other tissues. Salivary α-amylase initiates starch digestion in the oral cavity, and pancreatic α-amylase is the principal enzyme of its degradation in the small intestine. In mammalian genomes, α-amylases present multigene families, e.g., the human genome contains seven distinct amylase genes and also pseudogenes [20,21]. In humans, *AMY1* codes salivary α-amylase (human salivary α-amylase, HSA), *AMY2a*—pancreatic α-amylase (human pancreatic α-amylase, HPA) [22,23], and *AMY2b*—an isoform found in small intestine cells [24] and some tumors (specifically, lung carcinoma) [25,26].

Besides the conventional function of α-amylases, however, new findings suggest that their engagement in physiological processes may be broader. Notably, the expression level of *AMY2b* in the small intestine epithelium is second-highest after the pancreas. *AMY2b* knockdown suppressed the differentiation and proliferation of small intestine epithelium cells, which suggests a role of α-amylase in the process of their continuous renewal [24]. The human isoform AMY2A is reported to be involved in Alzheimer’s disease neuroinflammation. Its increased activity was discovered in the astrocytes of Alzheimer’s disease patients and is attributed to the reaction of cells to glycogen metabolism impairment and β-amyloid plaque formation [27,28].

## 3. Non-Proteinaceous α-Amylase Inhibitors

### 3.1. Pseudo-Oligosaccharides from Actinomycetes and Bacteria and Their Clinical Use

**Figure 2:**
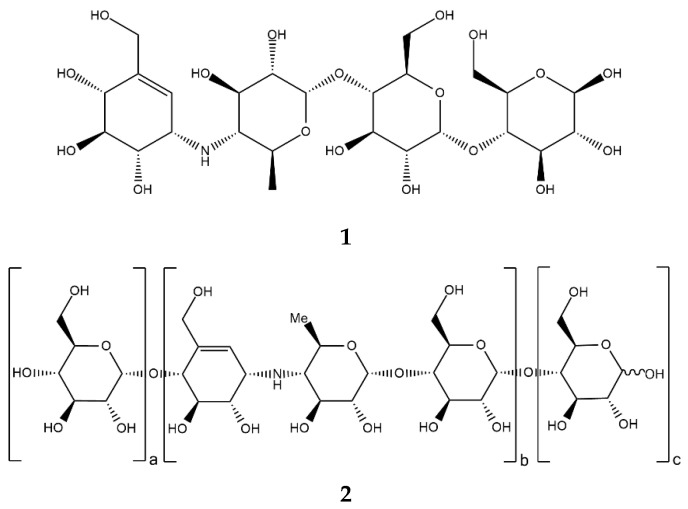
Chemical formulas of acarbose (**1**) and acarviostatins (**2**).

Acarbose (Figure 2, **1**), a clinically important drug that is used for the treatment of T2D, was originally found as a component of a complex mixture of pseudo-oligosaccharide-type secondary metabolites from actinomycete *Actinoplanes* strain SE 50/110. Structurally, acarbose contains a C7N aminocyclitol moiety (valienamine) linked via the nitrogen atom to 6-deoxyglucose (this dimer is also called acarviosin), which, in turn, is linked to a maltose moiety via an α-(1→4)-bond [29].

The properties of acarbose–enzyme interactions were studied through the analysis of the inhibitor–porcine pancreatic α-amylase (PPA) complex crystal structure [30]. The ability to inhibit PPA (*K*_i_ 0.8 µM [31]) is attributed, on the one hand, to the partial planarity of valienamine, which mimics the glucose residue transition state during the catalytic action of amylase, and, on the other hand, to the strong electrostatic interaction between carboxyl groups of the active site and a protonated amino group of the inhibitor [32].

In fact, numerous other acarviostatins (Figure 2, **2**) have been identified in bacterial isolates, and many of them are more potent α-amylase inhibitors than acarbose (e.g., acarviostatin III03 with *K*_i_ 0.008 µM) [33,34]. Each acarviostatin is composed of a pseudotrisaccharide core flanked by a variable number of D-glucose units.

Acarviostatin names are formed according to the following principle. The Roman numeral stands for the number of three-unit core repetitions, the middle Arabic digit corresponds to the number of D-glucose units at the non-reducing end, and the last Arabic digit is the number of D-glucose residues at the reducing end. According to this rule, another name for acarbose would be acarviostatin I01 [35].

**Figure 3:**
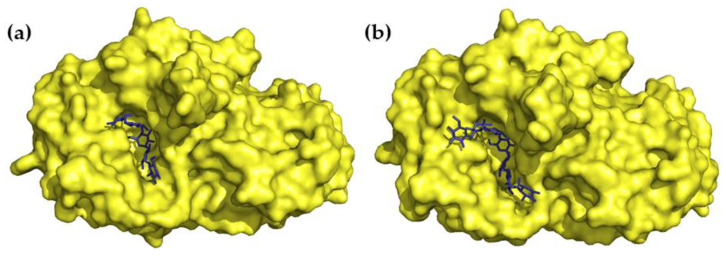
Three-dimensional structures of (**a**) acarbose in complex with HPA (PDB 1B2Y) and (**b**) the hydrolysis product of acarviostatin III03 in complex with HPA (PDB 3OLG).

Crystal structures of HPA in a complex with a series of acarviostatins showed that, in the same manner as acarbose, they all bind to the active site of HPA, and apparently, larger acarviostatins undergo hydrolysis at either one or both ends to generate acarviostatin II01. This suggests that an inhibitor containing seven sugar residues would provide the most efficient α-amylase inhibition through the complete occupation of the active site [36]. In all cases, transition-state mimicry underlies the inhibition mechanism. The nitrogen bond between valienamine and 6-dideoxyglucose is resistant to cleavage, which makes the acarviosin-containing compounds competitive inhibitors of mammalian α-amylases (Figure 3) [29,35].

**Figure 4:**
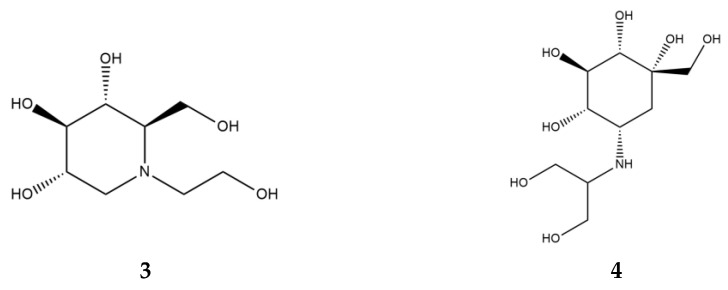
Chemical formulas of miglitol (**3**) and voglibose (**4**).

α-Glucosidase inhibitors (AGI), specifically, α-amylase inhibitors, are a distinct class of anti-diabetic drugs, but only three compounds (acarbose, miglitol (Figure 4, **3**), and voglibose (Figure 4, **4**)) are used in clinics and are commercially available. Acarbose originated as a natural product from the secondary metabolome of *Actinoplanes*, miglitol is a semisynthetic 1-deoxynojirimycin derivative, and voglibose is produced through the chemical modification of antibiotic validamycin C. Acarbose acts on α-amylase and other α-glucosidases, while voglibose and miglitol are effective against small intestine villi α-glucosidases but are inactive against starch-degrading α-amylase [37,38].

Acarbose, miglitol, and voglibose have been approved for usage by various national agencies, and acarbose is used most commonly. They are acknowledged as drugs with proven efficacy without any major safety concern and can be used as monotherapy or in combination with other oral hypoglycemic agents (sulfonylureas, metformin) or insulin. The therapeutic dose for acarbose and miglitol is 25 mg thrice daily with a gradual increase to 100 mg, administered at the start of each meal. The therapeutic dose of voglibose is 0.2 mg with a permissible increase to 0.3 mg, taken before or with a meal. The efficacy and tolerability of AGIs appear to be higher in Asia compared to Western countries, and this difference is likely due to the carbohydrate content of the diet. In addition to its improvement of glycemic parameters, this class of drugs also exerts beneficial effects on β-cell protection (through the control of postprandial hyperglycemia and decrease in glucose toxicity), body weight, and lipid metabolism. Cardiovascular benefits of AGI were also reported. The mechanisms behind them are multifactorial and apparently include reducing oxidative stress and inflammation, caused by postprandial hyperglycemia, and modulating gut hormones and the microbiota [37,38,39].

From a pharmacokinetic point of view, acarbose and voglibose are poorly absorbed and have low bioavailability. Miglitol, conversely, is almost completely absorbed in the jejunum. All three AGIs are distributed in the extracellular fluid, with low tissue affinity and variable protein binding. Acarbose and voglibose are metabolized principally by intestinal bacteria and are excreted via the fecal route, while miglitol is cleared unchanged by the kidneys. AGIs have few mild-to-moderate adverse effects, with no reported treatment-related toxicity. Undigested carbohydrates, when reaching the large intestine, can lead to flatulence, diarrhea, and abdominal pain. To reduce adverse effects, a small therapeutic dose with gradual increase is prescribed [38,40].

### 3.2. Secondary Metabolites from Plants and Prospects for Their Therapeutic Use

The scientific literature offers a plethora of reports concerning the in vitro α-glucosidase- and α-amylase-inhibitory activity of plant extracts used as anti-diabetic remedies [41,42,43,44]. Hypoglycemic activity is attributed to more than 1200 plant species, the usage of which has been recorded worldwide [45]. Extracts that show α-amylase and α-glucosidase inhibition in vitro comprise extensive lists of natural products incorporated into human diets. They embrace a diversity of fruit, vegetables, and mushrooms, e.g., citrus, pomegranates, berries, *Prunus* sp., onions, peppers, eggplants, bitter melons, and brassicas. Their effects are due to the ambiguous pool of natural compounds, including flavonoids, phenolic acids, anthocyanins, saponins, carotenoids, terpenes, sugars, proteins, capsaicinoids, fatty acids, and alkaloids [43,44,46].

**Figure 5:**
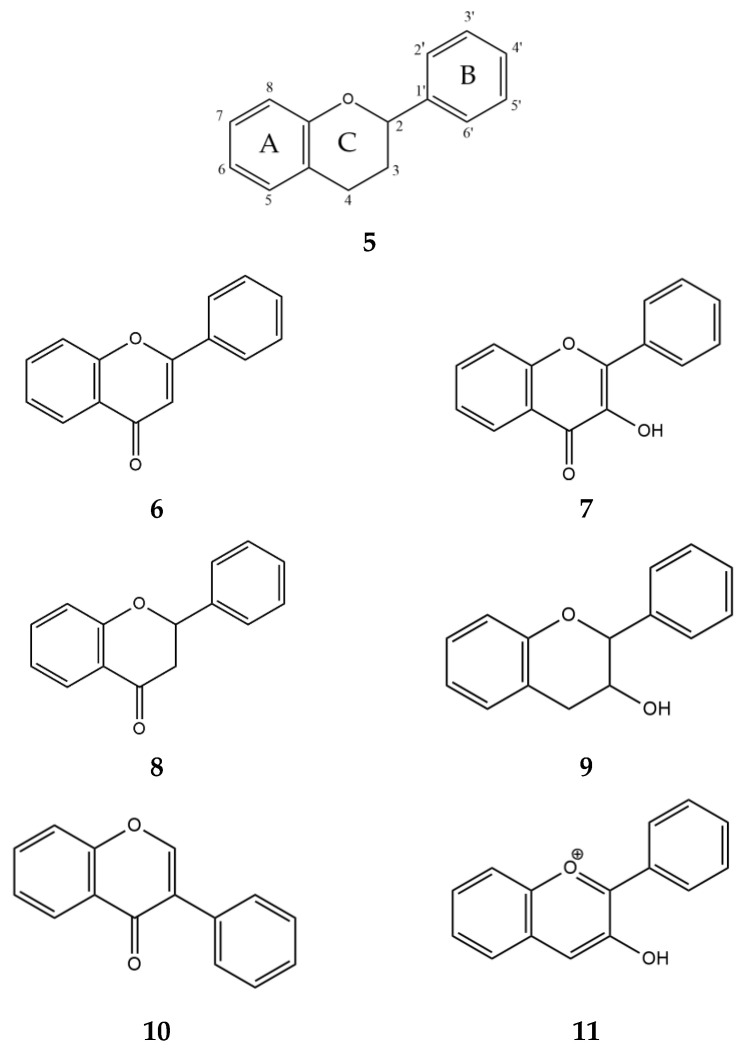
Generalized chemical formulas of flavonoids (**5**) and six subclasses of flavonoids: flavones (**6**), flavonols (**7**), flavanones (**8**), flavan-3-ols (**9**), isoflavones (**10**), and anthocyanidins (**11**).

Two classes of compounds, flavonoids and tannins, are known to inhibit α-glucosidase and α-amylase and play an important role in carbohydrate metabolism [47]. The most abundant secondary metabolites found in plants are flavonoids [44]. Structurally, they consist of two phenyl rings, A and B, linked by a three-carbon chain that forms an oxygenated heterocyclic C ring (Figure 5, **5**). There are six subclasses of flavonoids, including flavones (**6**), flavonols (**7**), flavanones (**8**), flavan-3-ols (**9**), isoflavones (**10**), and anthocyanidins (**11**), based on differences in the structure of the C ring, functional groups on the rings, and the position at which the B ring is attached to the C ring [48].

Naturally occurring flavonoids from the extracts of coffee, guava, whortleberry, olive, propolis, chocolate, and cocoa have been proposed as anti-diabetic and anti-obesity agents due to their various health benefits. Flavonoids’ effects on glucose and lipid metabolism are generally associated with modulating cellular signal pathways, receptors, and transporters. In particular, anti-diabetic and anti-obesity dietary flavonoids have been reported to regulate glucose transporters by increasing insulin secretion, reducing apoptosis, promoting pancreatic β-cell proliferation, and reducing insulin resistance, inflammation, and oxidative stress in muscles [49,50]. At the same time, no fewer than 500 flavonoid compounds have been reported to display α-amylase- or α-glucosidase-inhibitory activity, which demonstrates their value in the search for novel safer alternatives for postprandial hyperglycemic control [51].

Several productive attempts have been made to systemize knowledge on the α-amylase-inhibitory effects of flavonoids and provide insights into their structure–activity relationships [48,51,52,53,54,55]. Zhu et al. summarized data on different compounds from various reports, introducing a coefficient of efficacy relative to acarbose. This approach assisted in finding a positive correlation between the number of hydrogen bond donors and acceptors and the percentage of human α-amylase inhibition. Also, two modes of flavonoid-enzyme interactions were observed: (i) direct binding to the active sites of enzymes with the exclusion of substrate binding; (ii) an allosteric interaction near the active site and close to it. Some functional groups were correlated with stronger inhibitory effects: (i) substitutions of caffeoyl, galloyl, and prenyl groups enhanced the inhibitory effects; (ii) steric hindrance attenuated the inhibitory effects, and linear molecules tended to be stronger inhibitors of PPA. However, it is worth noting that a great hindrance to integrating data from different sources was inconsistent assay conditions, because the IC_50_ values for inhibitors are highly dependent on the enzyme concentration and origin, substrate type and concentration, reaction duration, temperature, and pH [48]. Xiao et al. focused the scope of their review on α-amylase inhibition and also inferred further general tendencies: (i) the presence of an unsaturated 2,3-bond in conjugation with a 4-carbonyl group accompanied stronger inhibition; (ii) the glycosylation of flavonoids reduced the inhibitory effect on α-amylase depending on the conjugation site and the class of sugar moiety; (iii) the methylation and methoxylation of flavonoids weakened the inhibitory effect [53].

Tannins are another heterogeneous group of polyphenols that is abundant in plants. They have a relatively high molecular weight, from 500 to 3000 Da, and can be divided into two major classes: hydrolysable tannins, which consist of esters of gallic acid (i.e., gallotannins) and ellagic acid (i.e., ellagitannins), and condensed tannins, which are also called proanthocyanidins [45,46,56].

Tannins are beneficial as plant food constituents due to their ability to reduce oxidative stress and cut down the incidence of diabetes, cancers, and cardiovascular diseases [57]. Their physiological effects on human health can also be attributed to their metal ion-chelating, antioxidant, and protein-precipitating properties [45,56,58]. The ability to strongly bind to proteins, forming insoluble and indigestible complexes, is probably the action mechanism that underlies α-amylase inhibition [58]. Kandra et al. also suggested that the interaction between galloylated quinic acid tannins and human salivary α-amylase may be facilitated by free OH groups in the tannins, which are able to participate in hydrogen bonding [59]. McDougall et al. showed that strawberry and raspberry extracts, which contained appreciable amounts of soluble tannins, effectively inhibited α-amylase. Other tannin-rich extracts (red grape, red wine, and green tea) were also effective inhibitors of α-amylase. Indeed, removing tannins from strawberry extracts with gelatin also removed inhibition. The inhibitory components were identified as ellagitannins. However, the extent of α-glucosidase inhibition was related to anthocyanin content. So, apparently, it is the synergistic action of different polyphenol components that modulates starch digestion rather than the action of an individual compound or a class [60].

There is increasing interest in indigenous medicinal herbs and natural dietary supplements that have been used for treating diabetes. Studies reporting their anti-diabetic effects regard ancient practices of Chinese, Japanese, Korean, Indian, Arabic, and African medicine [61].

In traditional Chinese medicine, there are 61 patented compositions and 166 patents for herbal extracts and their derivatives that are registered as oral antihyperglycemic therapeutics [62]. No fewer than 50 anti-diabetic formulas are approved for commercial use by the State Food and Drugs Administration of China [63,64]. Wang et al. listed 20 traditional Chinese herbs with reported α-glucosidase-inhibitory activity [63]. The long-term use of these agents may be advantageous over chemical drugs in alleviating some of the chronic conditions and complications caused by diabetes [65]. In particular, α-amylase inhibition was linked to the anti-diabetic activity of some traditional Chinese preparations. The widely known traditional herbal medicine *Polygonatum odoratum,* in the form of a total flavonoid extract, demonstrated a hypoglycemic effect on streptozotocin-induced diabetic mice and alloxan-induced diabetic rats, and the effect was accompanied by dose-dependent α-amylase inhibition in vitro [66].

In Ayurvedic medicine, over 800 plant species have been reported to be potential anti-diabetic drug sources [61,67]. Apparently, α-amylase inhibition underlies the action mechanism of some of them. Effective α-amylase inhibition was found in plant extracts of *Linum usitatisumum*, *Morus alba*, *Ocimum tenuiflorum*, *Curcuma longa*, *Cinnamonum verum*, *Ficus bengalensis*, *Syzygium cumini*, *Bixa orellana*, *Murraya koenigii*, and *Tribulus terrestris* [68,69].

Traditional Arabic medical practices also employ preparations for diabetes treatment. Several extracts subjected to in vitro and in vivo assays showed α-amylase- and α-glucosidase-inhibitory activities, including *Geranium graveolens*, *Varthemia iphionoides*, *Pistacia atlantica*, *Rheum ribes*, *Sarcopoterium spinosum*, *Arum dioscoridis*, and *Arum palaestinum* [70,71,72].

Dental caries and periodontitis are the most common infectious oral cavity diseases, and cause significant damage to human health because of the lack of timely treatment. Despite being largely preventable, dental diseases are highly prevalent conditions, affecting more than 3.5 billion people around the world. As with most non-communicable diseases, oral conditions are chronic and strongly socially patterned [73,74]. Dental caries develops due to microbial biofilm (plaque), which releases free acids in the process of sugar metabolism, dissolving hard tooth tissues (enamel and dentine). In the absence of proper oral hygiene, the disease course includes the formation of cavities in the teeth, occurrence of pain, and, in later stages, tooth loss and generalized infection. In periodontitis, inflammation occurs in the tissues surrounding and supporting the tooth. Periodontitis in the first stages is accompanied by bleeding and swelling of the gums, pain, bad breath, and, in later stages, tooth loss. A significant role in dental caries development is played by streptococci (*Streptococcus*) that inhabit the oral cavity [75]. Bacteria, i.e., *S. mitis*, *S. gordonii*, *S. salivarius*, *S. cristatus*, and *S. mutans*, are capable of binding HSA and utilizing the digested starch for their own needs. The binding of HSA by streptococci contributes to biofilm formation and dental demineralization [76].

Although a role of HSA in dental caries and periodontal disease is possible, currently, the drug market provides no medications for the treatment and prevention of these conditions, which are based on inhibiting HSA. Yet, several clinical trials of herbal preparations with high therapeutic potential are reported. A comparative in vivo study of black tea decoction in high- and low-caries children showed that it can significantly inhibit HSA activity irrespective of the caries index [77]. Another clinical study showed that black tea decoction possessed higher inhibitory activity than green tea decoction. The removal of tannins from the investigated preparations led to a complete loss of activity against HSA, and fluoride ions had no effect on the enzyme [78]. A handful of other studies reported that tea leaves and cherry extracts used as chewing gums can impede the growth of oral cavity streptococci (i.e., *Streptococcus mutans*) through the inactivation of HSA [79,80,81].

Plant extracts, as well as bioactive compounds of plant origin, employed as agents for α-amylase inhibition with hypoglycemic action can significantly contribute to the treatment of T2D and its complications. Although it is an attractive object of research and the information on the use of plant extracts and individual compounds in anti-diabetic therapy is abundant, there are still gaps in this field. Low-molecular inhibitors usually show moderate affinity for α-amylases (IC_50_ in the micromolar range). Also, additional in vivo tests are needed to study the pharmacological activity and synergistic interaction with other molecules for safer use.

## 4. Proteinaceous α-Amylase Inhibitors

### 4.1. Inhibitors from Streptomyces

Effective proteinaceous inhibitors of mammalian and bacterial α-amylases were found in bacteria of the *Streptomyces* genus: HOE 467 (tendamistat) from *S. tendae* [82], Z-2685 (parvulustat) from *S. parvulus* [83], Haim II from *S. griseosporeus* [84], AI-3688 from *S. aureofaciens* [85], Paim I from *S. corchorushii* [86], AI-409 from *S. chartreuses* [87], and T-76 from *S. nitrosporeus* [88]. These inhibitors have similar characteristics, e.g., the amino acid sequence length (around 75 a.a., 8 kDa), the β-barrel tertiary structure containing two β-sheets formed of two β-strands each, and the conserved position of two disulfide bonds. The reactive site contains the triad Trp18, Arg19, and Tyr20 and tightly binds in an equimolar complex with α-amylase. These amino acids are located on the β-loop on the surface of the proteins so that the hydrophobic part of the arginine residue is held between two stacked aromatic side-chains with the guanidinium group exposed to water [89,90,91,92,93].

**Figure 6:**
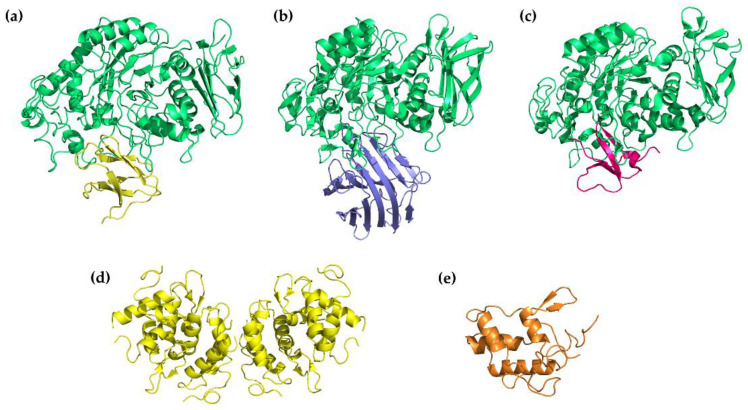
Three-dimensional structures of α-amylase inhibitors: (**a**) tendamistat from *Streptomyces tendae* (yellow) in a complex with PPA (green) (PDB 1BVN), (**b**) α-AI-1 from bean *Phaseolus vulgaris* (blue) in a complex with PPA (green) (PDB 1DHK), (**c**) helianthamide from sea anemone *Stichodactyla helianthus* (magenta) in a complex with PPA (green) (PDB 4X0N), (**d**) 0.19 α-amylase inhibitor (0.19 AI) from wheat *Triticum aestivum* (yellow) (PDB 1HSS), (**e**) RBI from Indian finger millet (ragi) (orange) (PDB 1BIP).

A three-dimensional structure of the tendamistat–PPA complex has been elucidated via X-ray analysis. It was shown that 30% of the water-accessible surface of the tendamistat contacts PPA. Four segments of the inhibitor’s polypeptide chain, with a total of 15 a.a., are involved in the complex formation. The segment, which carries the staggered side chains of the triad of Trp18, Arg19, and Tyr20, binds to the catalytic site with the formation of a salt bridge between the inhibitor’s Arg19 and the enzyme’s Glu230. Through hydrogen bonds and hydrophobic interactions, other segments fill the PPA’s groove, which is a substrate-binding region and also harbors acarbose [94]. The extended area of interaction between tendamistat and α-amylase explains the very low inhibition constant (*K*_i_ 9–200 pM) (Figure 6a) [95].

Tendamistat underwent clinical trials and showed the almost complete inactivation of salivary enzymes [96], the inhibition of starch absorption, and a significant decrease in blood glucose levels [97]. However, further investigation was quickly halted due to its susceptibility to degradation and remarkable immunogenicity [95,98].

An intriguing problem was why *Streptomyces* produce proteins that effectively and selectively inhibit mammalian α-amylases. It was speculated that tendamistat and its related inhibitors play a regulatory role because they tightly and irreversibly bind α-amylases of *Streptomyces*, as well [95]. The alignment of amino acid sequences showed that *S. limosus* α-amylase has a substantial (over 35%) identity with animal pancreatic α-amylases (*Mus musculus*, *Rattus norvegicus*, *Sus scrofa*, and *Drosophila melanogaster*), but low overall similarity with bacterial (*Bacillus subtilis*, 22.6%; *B. amyloliquefaciens*, 16.1%), fungal (*Aspergillus oryzae*, 20.1%), and plant (*Hordeum vulgare*, 21.7%) α-amylases. The elucidated tendency correlated well with the specificity of tendamistat [99]. Further studies on α-amylase’s molecular evolution supported this finding and combined α-amylases of animals and streptomycetes into one phylogenetic group [12].

### 4.2. Inhibitors from Plants

Numerous proteinaceous α-amylase inhibitors were isolated from plants. On the base of their tertiary structure, they are divided into six classes: knottin-like, legume lectin-like, cereal-type, Kunitz-like, γ-purothionin-like, and thaumatin-like. With several exceptions, all of them are highly specific to plant and insect amylases, since they participate in starch storage control and defense against pests. Among them, the number of proteins that act on mammalian α-amylases is rather limited and, in general, these targets are not primal in their broad inhibition spectra [100,101,102].

The family of lectin-like inhibitors is abundant in *Phaseolus* sp. [103]. The best studied isoform, α-AI1 (22.5 kDa), was purified from common bean *Phaseolus vulgaris* and was characterized at the molecular level as an α-amylase inhibitor of a novel class homologous to phytohemagglutinin [104]. α-AI1 potently inhibited PPA (*K*_i_ 3 × 10^−11^ M) [105,106] as well as insect α-amylases [100]. Another inhibitor, α-AI2, was isolated from wild accessions of the common bean. Although it shares 78% sequence similarity with α-AI1, it shows no activity towards mammalian α-amylases [107]. These inhibitors have a heterotetramer structure consisting of two pairs of noncovalently bound glycopeptide subunits α and β. α-AI1 and α-AI2 acquire their active form through a series of post-translational modifications. The polypeptide precursor is processed into two chains, which is followed by clipping the C-terminal Asn of the α-chain and seven C-terminal residues of the β-chain. The mature proteins also undergo glycosylation with different patterns for α-AI1 and α-AI2 [108]. The X-ray structures of α-AI1 in complex with PPA and HPA have been determined (Figure 6b). It was shown that the interaction occurs through the blockade of the enzyme’s catalytic residues with the inhibitor’s two hairpin loops extending out from the jellyroll fold. α-AI1 shows substrate mimicry, which allows it to effectively fill the substrate-docking region of the α-amylase. The inhibition of PPA and HPA is very similar, though it was revealed that α-AI1 forms additional hydrogen bonds with the B-domain of HPA in the region where amino acid sequences of the two enzymes differ [109,110]. Homology modeling and docking simulation predicted the structural determinants responsible for the different specificity of α-AI1 and α-AI2. The modeled α-AI2 did not meet the steric obstacles to penetrating the PPA cleft, but was unable to form enough hydrogen bonds for a stable complex due to amino acid sequence divergence from α-AI1 in the loop regions [111].

Cereal-type α-amylase inhibitors are found in wheat, barley, rye, and Indian finger millet (ragi). They are composed of 120–160 amino acid residues, contain five disulfide bonds, and vary in their specificity of action [100]. Wheat *Triticum aestivum* preparations contain several α-amylase inhibitors, which are coded after their gel-electrophoretic mobility relative to bromophenol blue: 0.19, 0.28, 0.53, etc. [112]. Among them, 0.19 α-amylase inhibitor (0.19 AI, 13.3 kDa) shows high-affinity inhibition of mammalian α-amylases (*K*_i_ 0.29 nM against HSA); however, it also acts on bird, Bacilli, and insect α-amylases [100,113,114]. The bifunctional α-amylase/trypsin inhibitor from Indian finger millet (ragi), RBI (13.1 kDa), is another prominent protein of the cereal-type family that acts potently, but also nonspecifically, on mammalian α-amylases (*K*_i_ 0.18 nM against PPA) [115]. Three-dimensional structures of these inhibitors were elucidated via X-ray and NMR spectroscopy (Figure 6d,e). RBI and 0.19 AI (26% sequence identity) both contain five disulfide bonds and share a common spatial arrangement of α-helices in an up-and-down manner. However, 0.19 AI contains five α-helices and two short antiparallel β-strands and is a homodimer, while RBI has four α-helices and a small antiparallel β-sheet and is monomeric [116,117]. Structural dissection of RBI showed that the sites responsible for the interaction with α-amylase and trypsin are independent, with the former residing in the N-terminal region. The α-amylase inhibition kinetics was shown to be complex and substrate-dependent. Although a separate N-terminal peptide (10 a.a.) and its mutant analogs showed regular competitive inhibition, integral RBI was observed to bind to the substrate [118].

Several clinical trials were devoted to the potential of wheat proteinaceous α-amylase inhibitor preparations in managing T2D. In healthy and diabetic subjects, these dietary supplements showed some credible therapeutic effects since they delayed carbohydrate absorption and decreased postprandial plasma glucose concentrations [119,120]. However, later findings call into question the prospects of their therapeutic use. In addition to the well-known fact that these inhibitors are associated with baker’s asthma and allergy to cereal flour [121], cereal α-amylase/trypsin inhibitors were found to elicit small intestinal inflammation in patients with celiac disease. Due to their ability to avoid proteolytic digestion by gastric and enteric proteases, they descend into the intestinal lumen where they can activate Toll-like receptor 4, which results in the release of proinflammatory cytokines. These strong innate immune effects were shown in vitro and in vivo after oral and systemic challenge. The observed effects may also have implications for intestinal inflammatory disorders of the gastrointestinal tract other than celiac disease [122,123,124].

Common bean extracts are used as dietary supplements for carbohydrate control. Specifically, Phase 2^®^ has two permitted structure/function claims: assistance in weight control when used in conjunction with diet and exercise, and a reduction in the enzymatic digestion of dietary starches. This preparation received GRAS (“generally recognized as safe”) status from the FDA, and the safety statements were supported by toxicity trials [125].

In multiple clinical trials, common bean extracts were shown to be effective in inhibiting α-amylase activity, decreasing postprandial blood glucose and insulin levels, and reducing body fat, but the results depended on the methods of manufacture and extraction [126]. Experiments, in which Phase 2 was incorporated into food and beverage products, have found that it can be integrated into various products without losing activity or altering the appearance, texture, or taste of the food [127]. An anorexigenic effect was suggested as a basis for obesity reduction, but its mechanism is not clearly understood. Apparently, the effect can only be achieved with prolonged exposure to the inhibitor, as was shown with Sprague Dawley rats [128]. Also, in a model of high-fat mice, the treatment with the standardized extract of *P. vulgaris* showed a significant reduction in several pathological features related to a metabolic-syndrome-like condition. Bean extract normalized the diet-evoked tolerance to glucose and insulin, decreased hepatic steatosis and lipid peroxidation in the liver, and protected the heart from oxidative alterations [129].

Obiro et al. also suggest the potential of common bean α-amylase inhibitor extracts for the prevention of colorectal cancer. The presence of the amylase inhibitor in the gut could cause an action similar to that of resistant starch, which acts as a prebiotic. The associated butyrate production halters malignant proliferation and leads to a reduced incidence of colon cancer. Although an increased ratio of butyrate/short-chain fatty acids, which inversely correlated with biomarkers of colonic neoplasia, was found after acarbose administration, such effects of common bean extracts are a probable subject of future research [126].

### 4.3. Inhibitors from Sea Anemones

Among animals, α-amylase inhibitors were found only in sea anemones (Cnidaria, Actiniaria). The first member of a novel group of α-amylase inhibitors belonging to a β-defensin family, helianthamide (4.7 kDa), was isolated from *Stichodactyla helianthus* [130]. This inhibitor acts specifically on mammalian α-amylases (*K*_i_ 0.01 nM against HPA and 0.1 nM against PPA) and is not active against human maltase–glucoamylase and bacterial α-amylases. Helianthamide is remarkably potent and, seemingly, is one of the smallest known natural proteinaceous inhibitors of mammalian α-amylases. This, as well as its compact structure and pronounced stability, highlights it among other peptide drug candidates (e.g., tendamistat).

Polypeptides that adopt a tertiary structure close to β-defensins were found in various organisms. Individual representatives of this family consist of 35–50 a.a. and interact with entirely different molecular targets, thus displaying a broad spectrum of biological activity. The structures of the β-defensin fold family commonly contain a short helix or a turn followed by a small twisted antiparallel β-sheet. Six cysteine residues which are paired in a 1–5, 2–4, 3–6 manner are key to sustaining the compact configuration of these structures [131,132].

A crystal of PPA in the complex with linear chemically synthesized helianthamide with reduced disulfide bonds was obtained (Figure 6c) [130]. The structural model showed that helianthamide forms a noncovalent complex with the enzyme. One third of the water-accessible surface of helianthamide contacts PPA, mainly, directly in the active site and near it. A new YIYH-inhibitory motif of helianthamide was proposed to account for the interaction with α-amylase active site residues. Later, it was demonstrated that the alanine and phenylalanine substitutions of the polar residues in the determined motif reduced the binding affinity only 5–46-fold compared to the wild-type helianthamide, which indicates a rather modest contribution of the YIYH motif in the interaction with the enzyme. On the contrary, individual substitutions of six cysteines to alanine lead to a sufficiently greater decrease in the inhibitory activity of mutants (up to four orders compared to the wild type). Additionally, on the basis of helianthamide, an array of smaller peptides was synthesized and tested. Only 2 out of 19 peptides showed any substantial affinity for HPA, with IC_50_ 141 and 396 μM, which is seven orders greater than the inhibition constant of wild-type helianthamide (*K*_i_ 0.01 nM). These results show that the high inhibitory potency of helianthamide is not determined by individual polar contacts but by its ability to form an extended hydrophobic interface and occlude the active site cleft [133].

Proteomic analysis of sea anemone *Heteractis magnifica* mucus revealed multiple isoforms of α-amylase inhibitors [134]. The major inhibitor magnificamide (4.7 kDa) was isolated and characterized. Magnificamide shared 84% amino acid sequence identity with helianthamide and potently inhibited PPA (*K*_i_ 0.17 nM), HSA (*K*_i_ 7.7 nM) [135], and HPA (*K*_i_ 3.1 nM) [136]. The analysis of the impact of heating on the spatial structure and biological activity of magnificamide demonstrated its incredible thermostability. Magnificamide inhibited PPA completely in a heating range up to 80 °C, while at temperatures up to 100 °C, its activity decreased only by 12% [136]. In electrophysiological testing on 12 subtypes of voltage-gated potassium and 6 subtypes of voltage-gated sodium channels, including heart channels (hERG), expressed in *Xenopus laevis* oocytes, magnificamide showed no modulation of these types of ion channels [135]. It neither showed toxicity when administered orally and intravenously at a dose of 2 mg/kg, nor had any negative effect on the central nervous systems of mice [136]. Also, an in vivo study on a model of streptozotocin-induced type 1 diabetes mellitus, it was shown that magnificamide at a dose of 0.005 mg/kg was able to suppress postprandial hyperglycemia more effectively than acarbose at a dose of 3 mg/kg.

Proteinaceous and some representatives of non-proteinaceous inhibitors of mammalian α-amylases discussed in this review are summarized in Table 2.

Using the step-out RACE (rapid amplification of complementary ends) approach, in addition to magnificamide, six isoforms with point substitutions in mature peptides were found in *H. magnifica* tentacles [142]. According to the TBLASTN search among the transcriptomes of the *Actiniaria* order, other sea anemones also share a number of magnificamide-like transcripts. For the first time, the exon–intron structure of the magnificamide genes was established, and the phenomenon of intron retention in the region encoding the mature peptide was discovered, which probably leads to an additional variety of inhibitor isoforms and allows for their neofunctionalization. In addition, it was discovered for the first time that the domain responsible for the inhibition of α-amylase is also a part of mucins, protective glycoproteins of some sea anemones. The bioprospecting of sea anemones for the presence of β-defensin-like inhibitors revealed a variety of inhibitory sequences that could become a source for the development of novel hypoglycemic drugs.

## 5. Conclusions

As α-amylases are among the primal digestive enzymes in humans, they present an actual therapeutic target in disorders associated with excessive carbohydrate intake. Therapeutic oral α-glucosidase inhibitors target the salivary and pancreatic α-amylases and α-glucosidases of small intestine villi. Acarbose and miglitol as active ingredients of these anti-diabetic drugs are approved by FDA for the management of T2D and are effective for the control of postprandial hyperglycemia.

This review focused on the inhibitors of mammalian α-amylases. A major class of these active molecules includes pseudo-oligosaccharides that mimic substrates and block enzyme activity. Another major class includes plant metabolites, e.g., flavonoids and tannins, that display a variety of structural motifs and mechanisms of action, from non-competitive inhibition to nonselective protein binding. An intriguing class of active compounds is proteinaceous α-amylase inhibitors from bacterial, plant, and animal sources. Some of them have found applications as dietary supplements for weight control (kidney bean extracts). Inhibitors from sea anemones present high-affinity ligands of mammalian α-amylases, derived from animal venom but without apparent toxicity. The advantage of proteinaceous inhibitors belonging to the β-defensin family is their structural and physico-chemical characteristics, such as protein compact fold, resistance to proteolysis, and prolonged exposure to low pH values and high temperature.

Natural products are a prospective source of new lead compounds for pharmacy that present a rich pool of molecular scaffolds with varying selectivity and affinity. Many compounds that underlie the bioactivity of the extracts are still not known in individual form. Future studies are required for the development of extraction techniques, the preparation of α-glucosidase- and α-amylase-inhibitory fractions or individual lead compounds, and the examination of their therapeutic potential and probable toxicity and off-target activity.

At the early stages of drug development, the bioprospecting of biologically active natural products based on genomic/transcriptomic studies can be a valuable complementary tool. This direction may become promising in the search for new drugs for metabolic disease treatment.

## Figures and Tables

**Table 1:** Molecular features of mammalian α-amylases.

Mammal	Name	Sequence Identity, %	a.a.	Molecular Weight, kDa
Human	1A (salivary)	100	496	55.9
	2A (pancreatic)	97	496	55.9
	2B (carcinoid)	98	496	55.9
*Sus scrofa*	Pancreatic	86	496	55.4
*Mus musculus*	1 (salivary and liver)	85	496	55.9
	2 (pancreatic)	84	493	55.6
*Rattus norvegicus*	Pancreatic	84	493	55.5

**Table 2:** Proteinaceous and some representatives of non-proteinaceous inhibitors of mammalian α-amylases.

Structural Class/Origin	Source	Name	Target	IC_50_/*K*_i_, M	Ref.
Non-proteinaceous α-amylase inhibitors					
Actinomycete	*Actinoplanes* strain SE 50/110	Acarbose	PPA	0.8 × 10^−6^	[31]
Flavonoids	*Potentilla anserine* (rhizome)	Quercetin-3-O-α-L-rhamnopyranoside-2″-gallate	α-glucosidase, α-amylase	1.05 × 10^−6^	[137]
	*Knema globularia* (stem)	Calodenin A		0.4 × 10^−6^	[138]
	*Caesalpinia paraguariensis* (bark)	(−) epigallocatechin-gallate		5.20 × 10^−6^	[139]
Tannins	*Wendlandia glabrata*	Procyanidin A2		0.47 × 10^−6^	[140]
	*Rubus chingii* Hu	Chingiitannin A		4.52 × 10^−6^	
Proteinaceous α-amylase inhibitors					
Microbial	*Streptomyces tendae*	Tendamistat	HPA	9 × 10^−12^ – 2 × 10^−10^	[95]
	*Streptomyces parvulus*	Parvulustat	PPA	2.8 × 10^−11^	[141]
Legume lectin-like	*Phaseolus vulgaris*	α-AI1 (heterodimer)	PPA	3 × 10^−11^	[106]
			HPA	+	[110]
Cereal-type	*Triticum aestivum*	0.19 (monomer)	PPA	5.73 × 10^−8^	[113]
			HSA	2.9 × 10^−10^	[114]
	*Eleusine coracana*	RBI	PPA	1.8 × 10^−10^	[115]
β-defensin-like	*Stichodactyla helianthus*	Helianthamide	PPA	1 × 10^−10^	[130]
			HSA	1 × 10^−11^	[130]
	*Heteractis magnifica*	Magnificamide	PPA	1.7 × 10^−10^	[135]
			HSA	7.7 × 10^−9^	[135]
			HPA	3.1 × 10^−9^	[136]

## Data Availability

Data are contained within the article.
